# Exercise during chemotherapy: Friend or foe?

**DOI:** 10.1002/cam4.5831

**Published:** 2023-04-19

**Authors:** Melanie Potiaumpai, Erica A. Schleicher, Ming Wang, Kristin L. Campbell, Kathleen Sturgeon, Kathryn H. Schmitz

**Affiliations:** ^1^ Department of Public Health Sciences, College of Medicine Pennsylvania State University Hershey Pennsylvania USA; ^2^ Department of Health Behavior, School of Public Health University of Alabama at Birmingham Birmingham Alabama USA; ^3^ Department of Population and Quantitative Health Sciences, School of Medicine Case Western Reserve University Cleveland Ohio USA; ^4^ Department of Physical Therapy, Faculty of Medicine University of British Columbia Vancouver British Columbia Canada; ^5^ Department of Medicine University of Pittsburgh Pennsylvania Pittsburgh USA

**Keywords:** chemotherapy, chemotherapy completion, exercise, exercise oncology, relative dose intensity

## Abstract

**Background:**

A higher chemotherapy completion rate is associated with better outcomes including treatment efficacy and overall survival. Exercise may have the potential to improve relative dose intensity (RDI) by reducing the frequency and severity of chemotherapy‐related toxicities. We examined the association between exercise adherence and RDI and possible clinical‐ and health‐related fitness predictors of RDI.

**Methods:**

Chemotherapy records were extracted from the electronic medical record for patients enrolled in the ENACT trial (*n* = 105). Chemotherapy completion was assessed using average RDI. A threshold of 85% was established for “high” versus “low” RDI. Logistic regression analyses were used to estimate the associations between the clinical‐ and health‐related fitness predictors of RDI.

**Results:**

Patients with breast cancer (BC) had a significantly higher average RDI (89.8% ± 17.6%) compared with gastrointestinal cancer (GI) (76.8% ± 20.9%, *p* = 0.004) and pancreatic cancer (PC) (65.2% ± 20.1%, *p* < 0.001). Only 25% of BC patents required a dose reduction compared to 56.3% of GI and 86.4% of PC patients. Cancer site was significantly associated with RDI. Compared with BC, patients with GI (*β* = −0.12, *p* = 0.03) and PC (*β* = −0.22, *p* = 0.006) achieved significantly lower RDI. Every 2.72 unit increase in overall exercise adherence led to a significant 7% decrease in RDI (*p* = 0.001) in GI patients. Metastatic GI patients had a 15% RDI increase for every 2.72 unit increase in exercise adherence (*p* = 0.04).

**Conclusion:**

Exercise is a supportive therapy that has potential to enhance chemotherapy tolerance and completion. The relationship between exercise adherence and RDI is influenced by factor such as cancer site and treatment type. Special attention must be paid to how exercise is prescribed to ensure that exercise adherence does not negatively affect RDI. Cancer site, exercise dosage, and multimodal interventions to address toxicities are key areas identified for future research.

## Graphical abstract

Exercise during chemotherapy has the strong potential to reduce the frequency and intensity of chemotherapy‐related toxicities. The inclusion of exercise is safe and can support higher chemotherapy completion rates for patients receiving active treatment for breast, gastrointestinal, and pancreatic cancer.

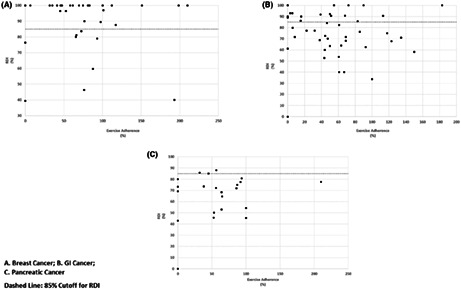

## 1 INTRODUCTION

In 2021, approximately half of the estimated 1.9 million new cancer cases in the United States were planned to receive chemotherapy.^1^, ^2^ Mounting evidence demonstrates the importance for patients to complete their prescribed chemotherapy treatment according to the planned schedule.^3^, ^4^, ^5^ Insufficient chemotherapy completion, typically reported as relative dose intensity (RDI) of <85%, is associated with reduced treatment efficacy, reduced progression‐free survival, and increased risk of all‐cause mortality.^6^, ^7^ Existing evidence reports that increasing age, obesity, and reduced ECOG physical performance status may be significant contributors to incidences of reduced RDI.^7^, ^8^

Exercise has been identified as a compelling supportive therapy that could help maximize RDI and reduce chemotherapy‐related toxicities.^9^, ^10^ In addition to improvements in physical and patient‐reported outcomes, exercise has the potential to address chemotherapy dosing factors such as weight, performance status, and comorbidities.^9^, ^10^ The accumulation of these potential benefits could help reduce healthcare utilization.^10^, ^11^

The role of fitness capacity as a surrogate marker of overall health is understudied in relationship to RDI. To our knowledge, there are only two studies that report predictors of chemotherapy completion related to exercise participation or fitness. An et al. found that patients who performed in the highest 20% of VO_2peak_ and highest 80% of chest strength were significantly more likely to achieve ≥85% RDI.^12^ Similarly, Groen et al. reported that lower pretreatment physical fitness was associated with lower odds of achieving ≥85%, even after correcting for age.^13^

Few studies have evaluated the effect of exercise on RDI in mixed cancer sites, rather, focusing on a single cancer site.^9^ However, cancer exercise physiologists routinely work with many cancer sites requiring studies that reflect the real‐world value and practical realities of working in a clinical cancer care setting. Therefore, our objective was a post hoc analysis of the ENACT trial to evaluate the association of exercise adherence with RDI. We were interested in the correlation between exercise adherence and RDI on a continuous basis, and describing the exercise adherence level associated with an RDI of >85%. Additionally, we performed an exploratory analysis of clinical‐ and health‐related fitness variables to understand predictors of high RDI in patients with breast (BC), gastrointestinal (GI), and pancreatic (PC) cancer.

## 2 METHODS

The ENACT trial was a mixed methods pre‐ and post‐ single group pragmatic trial to assess the feasibility and acceptability of embedding an exercise trainer into the chemotherapy infusion suite from the perspective of clinicians and patients at the Penn State Cancer Institute (PSCI) (NCT03461471).^14^ The Penn State Human Subjects Protection Office and Institutional Review Board approved this protocol, and all patients provided written consent prior to any study‐related activities.

### 2.1 Patient description

Patients had to be seen at PSCI for outpatient cancer infusion therapy, be 18 years of age or older, and be receiving infusion therapy for a solid tumor, regardless of stage of cancer. Patients were excluded if they were pregnant, if there was evidence in the medical record of an absolute contraindication for exercise, or if the medical oncologist and/or exercise trainer identified a diagnosis that would make unsupervised exercise unsafe.^14^ This post hoc analysis included patients actively receiving outpatient chemotherapy for BC, GI, or PC. We chose to focus on these diagnoses due to the larger available sample size for each cancer site allowing for a comprehensive look into the relationship between exercise and RDI.

### 2.2 Measurements

Chemotherapy regimens including chemotherapy type, dosages, and duration were abstracted from the electronic medical record. Each chemotherapy infusion was recorded to track reductions in dose, dose delays, or missed doses. Information was recorded on chemotherapy regimens based on the start date of when the patient was consented for participation in ENACT until the end of their primary treatment, coinciding with study duration of ENACT participation. Demographic and clinical information (i.e., cancer site, disease stage, and comorbidities) were previously gathered from the electronic medical record at the time of consent for ENACT participation.

### 2.3 Relative dose intensity

RDI is a commonly used summary measure to describe dose reductions and/or delays during chemotherapy treatment.^6^, ^15^ RDI is calculated as the ratio of delivered dose intensity (dose actually administered over chemotherapy course) to the standard dose intensity (standard dose prescribed over chemotherapy course), multiplied by 100 to calculate the percent RDI.^16^ A threshold of 85% was established for “high” versus “low” RDI.^9^ For multi‐agent chemotherapy regimens, RDI was calculated as a mean value of the individual RDIs from each agent in the regimen, which is the accepted methodology.^16^

A dose reduction was defined as a patient experiencing a reduction of ≥15% in chemotherapy dose for at least one agent in any chemotherapy cycle relative to the planned standard dose.^7^ A dose delay was identified if there was a delay of seven or more days for at least one agent in any chemotherapy cycle relative to the planned date of administration.^7^ A missing dose was identified if a patient did not receive at least one agent that was part of the planned standard chemotherapy regimen. A missing dose was considered both a dose delay and dose reduction for that cycle, which is the common approach.^7^

### 2.4 Exercise intervention

The exercise intervention has been previously described.^14^ Briefly, the main exercise prescription was home‐based resistance training, which specified frequency, intensity, and time for each exercise.^14^ Although resistance training was the main exercise prescription, aerobic exercise (ranging from 5 to 30 min/session) was incorporated for patients if they were deemed functionally capable. Patients were provided personalized exercise logs and an exercise manual to track their exercises at home between infusion visits. Due to the pragmatic nature of the ENACT trial, each patients’ exercise prescription was personalized based on different factors including baseline functionality and pre‐existing comorbidities and symptomology. As there are no formal guidelines for exercise during active treatment, we advised that patients strive to complete at least 2 days per week of resistance training, and to complete additional exercise sessions if they felt “able” to. At each infusion, an exercise and cancer specialist reviewed the exercises and provided any necessary modifications. As multiple cancer sites were included in ENACT, different treatment regimens were included, so patients met with the exercise and cancer specialist at different frequencies.

Exercise adherence was calculated as the proportion of completed exercise sessions (as indicated by completed and returned exercise logs) compared with the number of prescribed exercise sessions. An exercise session was considered complete if the patient was able to complete at least two prescribed exercises. We grouped patients into two groups based on their exercise adherence: <70% was considered low and ≥70% was considered high based on the median split.

### 2.5 Statistical analysis

Descriptive statistics for overall and stratified by cancer type (BC, GI, and PC) were presented as frequencies (percentage, %) for categorical variables and mean (standard deviations, SD) for continuous variables. The normality assumption for continuous variables was checked based on Shapiro–Wilk tests, and if failed, log‐transformation was applied (e.g., RDI). For group comparisons (cancer types; levels of exercise adherence) of categorical variables, chi‐squared tests or Fisher's exact tests were used. For continuous variables, the two‐sample *t*‐tests or Wilcoxon rank sum tests were used for two‐group comparisons, and the analysis of variance or the Kruskal–Wallis test were for three‐group comparisons, as appropriate. To further evaluate the association of exercise adherence with RDI, multivariable regressions were performed for all patients combined and each cancer type, where potential confounding variables including metastasis and the number of comorbidities were considered. The back‐transformed parameter estimates to the original scale of RDI with 95% confidence intervals and Wald test‐based *p*‐values were obtained. All hypothesis tests were two‐sided with the significance level of 0.05. Data were analyzed using R version 4.2.1.

## 3 RESULTS

### 3.1 Participant description

Table 1 presents participant characteristics. The final analysis included 105 participants. Participants were on average 58 years old, 70% women, were majority Caucasian (94%), nonmetastatic (58%), had an ECOG score ≤2, reported little‐to‐no pain, and the majority presented with one or more comorbidities at study start. Of 105 participants, 35 were diagnosed with BC (33%), 48 were diagnosed with GI (46%), and 22 were diagnosed with PC (21%). There were no significant differences between cancer sites for age, race, or pain. Over 50% of BC patients were Stages I–III and nonmetastatic, whereas over 70% of GI and PC patients were Stages III and IV and metastatic.

**TABLE 1:** Participant characteristics.

	Overall (*n* = 105)	Breast (*n* = 35)	GI (*n* = 48)	Pancreatic (*n* = 22)	*p*‐value
*Age* (*years*), *mean ± SD*	58.1 ± 11.6	54.8 ± 9.9	58.7 ± 12.1	62.1 ± 12.3	0.06
*Sex, N* (*%*)					
Female	73 (69.5)	35 (100)	27 (56.3)	11 (50.0)	<0.001
Male	32 (30.5)	0 (0)	21 (43.8)	11 (50.0)	
*Race, N* (*%*)					
White	99 (94.3)	33 (94.3)	45 (93.8)	21 (95.5)	0.86
Black	5 (4.8)	2 (5.7)	2 (4.2)	1 (4.5)	
Other	1 (10)	0 (0)	1 (2.1)	0 (0)	
*Stage* (*1–4*), *N* (*%*)					
1	11 (10.5)	7 (20.0)	2 (4.2)	2 (9.1)	<0.001
2	23 (21.9)	13 (37.1)	6 (12.5)	4 (18.2)	
3	28 (26.7)	9 (25.7)	16 (33.3)	3 (13.6)	
4	39 (37.1)	6 (17.1)	23 (47.9)	10 (45.5)	
Unknown	4 (3.8)	0 (0)	1 (2.1)	3 (13.6)	
*Metastatic status, N* (*%*)					
Non‐metastatic	61 (58.1)	28 (80.0)	22 (45.8)	11 (50.0)	0.005
Metastatic	44 (41.9)	7 (20.0)	26 (54.2)	11 (50.0)	
*ECOG* (*0–5*), *N* (*%*)					
0	69 (65.7)	26 (74.3)	31 (64.6)	12 (54.5)	0.10
1	23 (21.9)	5 (14.3)	13 (27.1)	5 (22.7)	
2	5 (4.8)	0 (0)	2 (4.2)	3 (13.6)	
Unknown	8 (7.6)	4 (11.4)	2 (4.2)	2 (9.1)	
*Pain* (*0–10*), *mean ± SD*	0.94 ± 1.92	0.82 ± 1.86	0.73 ± 1.94	1.68 ± 1.89	0.17
*Total # of comorbidities, mean ± SD; N* (*%*)	1.2 ± 1.3	0.7 ± 0.8	0.9 ± 0.9	2.5 ± 1.7	<0.001
0	41 (39.0)	17 (48.6)	22 (45.8)	2 (9.1)	0.002
1	28 (26.7)	11 (31.4)	12 (25.0)	5 (22.7)	
2+	36 (34.3)	7 (20.0)	14 (29.2)	15 (68.2)	

Abbreviation: GI; gastrointestinal.

### 3.2 Relative dose intensity, dose reductions, dose delays

Table 2 illustrates RDI across all patients and between cancer sites. Across all cancer sites, RDI was 78.7% ± 21.5% (mean ± SD). Average RDI for BC was 89.8% ± 17.6%, which was significantly higher than GI (*p* = 0.004) and PC (*p* < 0.001).

**TABLE 2:** Chemotherapy completion and modifications.

	All cancer sites (*n* = 105)	Breast (*n* = 35)	GI (*n* = 48)	Pancreatic (*n* = 22)	*p*‐value (breast vs. GI)	*p*‐value (breast vs. pancreatic)	*p*‐value (GI vs. pancreatic)
RDI, mean ± SD	78.7 ± 21.5	89.8 ± 17.6	76.8 ± 20.9	65.2 ± 20.1	0.004	<0.001	0.03
Dose reduction, *N* (%)	55 (52.4)	9 (25.7)	27 (56.3)	19 (86.4)	0.006	<0.001	0.01
RDI ≥85%, *N* (%)	50 (47.6)	26 (74.3)	21 (43.8)	3 (13.6)	0.006	<0.001	0.01
Dose delay, *N* (%)	26 (24.8)	3 (8.6)	16 (33.3)	7 (31.8)	0.009	0.03	0.90

Abbreviations: GI; gastrointestinal, RDI; relative dose intensity.

Overall, 52.4% (*n* = 55) of all patients required a dose reduction. A significantly lower proportion of patients with BC required a dose reduction (25.7%), compared with GI (*p* = 0.006) and PC (*p* < 0.001). Patients that required a dose reduction averaged a RDI of 63.0% ± 18.4% and an exercise adherence of 62.8% ± 46.3%, whereas patients that did not require a dose reduction averaged a RDI of 95.9% ± 5.3% and an exercise adherence of 57.5% ± 52.4%. In BC patients that required a dose reduction, mean RDI was 65.1% ± 18.8% and exercise adherence averaged was 72.6% ± 56.8%. In GI patients, RDI averaged 63.1% ± 17.9% and exercise adherence was 60.7% ± 39.8%. In PC, RDI averaged 61.9% ± 19.7% and exercise adherence averaged 61.1% ± 51.6%.

Additionally, 24.8% (*n* = 26) of all patients required a dose delay of 1.1 ± 2.2 weeks on average. In those that required a dose delay, RDI averaged 69.4% ± 16.3% and exercise adherence averaged 45.0% ± 38.7%. For BC patients, 8.6% experienced a dose delay averaging 0.5 ± 1.1 weeks, a RDI of 70.7% ± 28.9%, and exercise adherence of 15.0% ± 26.0%; 33.3% of GI patients experienced a dose delay averaging 1.2 ± 2.6 weeks, a RDI of 70.5% ± 14.7% and exercise adherence of 52.1% ± 41.7%; and 31.8% (*n* = 7) of PC patients experienced a dose delay averaging 1.2 ± 1.9 weeks, a RDI of 66.1% ± 16.3% and exercise adherence of 41.6% ± 32.7%.

### 3.3 Exercise adherence versus RDI

**FIGURE 1:**
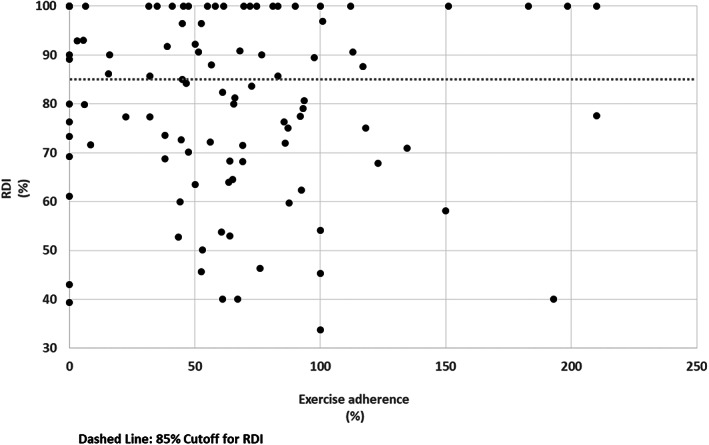
Exercise adherence versus relative dose intensity (RDI)—All patients.

**FIGURE 2:**
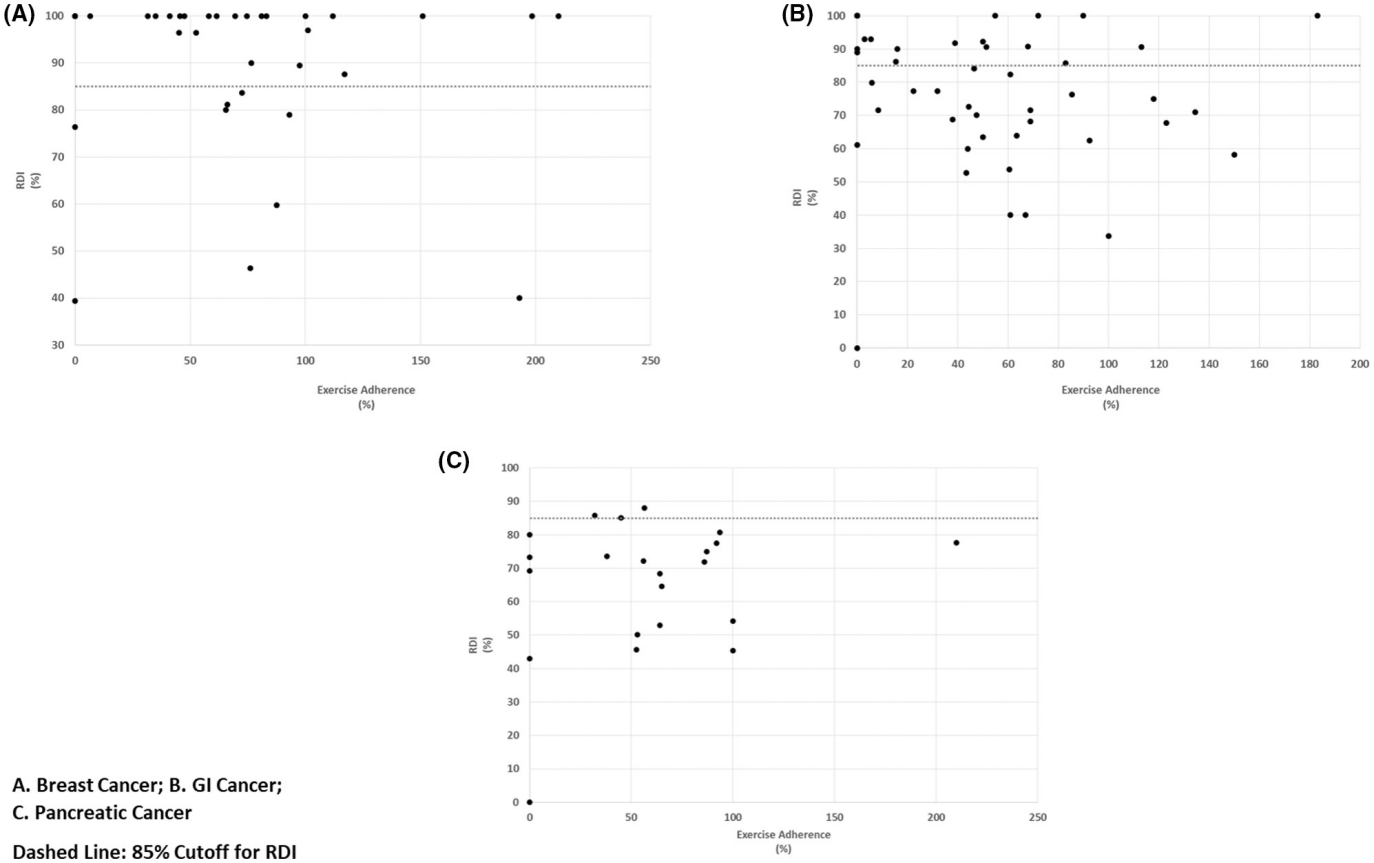
Exercise adherence versus relative dose intensity (RDI)—By cancer site. (A) Breast cancer. (B) GI cancer. (C) Pancreatic cancer. Dashed line indicates 85% cutoff for RDI.

Figure 1 illustrates the relationship between exercise adherence and RDI category for all patients. Although 34.3% of patients achieved 70% or higher exercise adherence, only 47.2% of these patients achieved a high RDI of ≥85%. Figure 2 illustrates RDI completion by cancer site. In BC, 74.3% (*n* = 26) of patients achieved a RDI of ≥85%, despite only 48.6% (*n* = 17) achieving ≥70% exercise adherence (Figure 2A). In GI, 43.8% (*n* = 21) of patients achieved a RDI of ≥85%, despite 25.0% (*n* = 12) achieving ≥70% exercise adherence (Figure 2B). In PC, only 13.6% (*n* = 3) of patients achieved a RDI of ≥85% and of the 31.8% (*n* = 7) of patients that achieved ≥70% exercise adherence, none of these patients achieved ≥85% RDI (Figure 2C).

### 3.4 Influence of exercise adherence on RDI

Comparison of the RDI between participants with low‐ versus high‐exercise adherence shows no significant difference (*p* = 0.67), and holds true across BC (*p* = 0.31), GI (*p* = 0.99), and PC (*p* = 0.49) (Table 3). In all patients, there was no difference in average RDI in between nonadvanced, low‐exercise adherence participants (83.8% ± 24.6%) compared with non‐advanced, high‐exercise adherence participants (83.4% ± 19.4%; *p* = 0.95) or in advanced, low‐exercise adherence (75.7% ± 21.5%) participants compared with advanced, high‐exercise adherence (77.7% ± 19.8%; *p* = 0.72). There was also no difference in RDI for each cancer site for nonadvanced, low‐ versus high‐exercise adherence or for advanced, low‐ versus high‐exercise adherence (Table 3).

**TABLE 3:** Relative dose intensity (RDI) by cancer site, advanced stage status, and exercise adherence.

	Low exercise adherence (*n* = 69)	High exercise adherence (*n* = 36)	*p*‐value	Nonadvanced, low adherence (*n* = 20)	Nonadvanced, high adherence (*n* = 14)	*p*‐value	Advanced, low adherence (*n* = 49)	Advanced, high adherence (*n* = 22)	*p*‐value
Overall (*N* = 105)	78.1 ± 22.5	79.9 ± 19.6	0.67	83.8 ± 24.6	83.4 ± 19.4	0.95	75.7 ± 21.5	77.6 ± 19.8	0.72
Breast	92.8 ± 15.5	86.6 ± 19.6	0.31	95.3 ± 8.4	83.3 ± 21.2	0.13	90.2 ± 20.6	92.8 ± 16.2	0.80
(*n* = 35)	(*n* = 18)	(*n* = 17)		(*n* = 9)	(*n* = 11)		(*n* = 9)	(*n* = 6)	
GI	76.8 ± 21.5	76.7 ± 20.0	0.99	75.9 ± 39.8	88.1 ± 16.8	0.70	76.9 ± 16.8	74.4 ± 20.6	0.70
(*n* = 48)	(*n* = 36)	(*n* = 12)		(*n* = 6)	(*n* = 2)		(*n* = 30)	(*n* = 10)	
Pancreatic	63.4 ± 22.7	68.9 ± 13.6	0.49	72.7 ± 14.2	75.0 ± 0.0	0.89	58.8 ± 25.3	67.9 ± 14.6	0.44
(*n* = 22)	(*n* = 15)	(*n* = 7)		(*n* = 5)	(*n* = 1)		(*n* = 10)	(*n* = 6)	

*Note*: Values are mean ± SD. Non‐advanced: Stages 1 and 2; advanced: Stages 3 and 4. Low exercise adherence: <70%; high exercise adherence: ≥70%.

Abbreviation: GI; gastrointestinal.

### 3.5 Dose delays and reductions

There was no difference in the proportion of patients who achieved a high RDI in the low‐ versus high‐exercise adherence groups (*p* = 0.95). The level of exercise adherence (low vs. high) did not have a significant effect on whether a patient received a dose reduction for BC (22.2% vs. 29.4%, *p* = 0.63), GI (55.6% vs. 58.3%, *p* = 0.87), or PC patients (80% vs. 100%, *p* = 0.20).

The proportion of patients who received a dose reduction in nonadvanced, low‐ versus high‐exercise adherence did not significantly differ for BC (22.2% vs. 36.4%, *p* = 0.50), GI (33.3% vs. 33.3%, *p* = 1.00), or PC (40% vs. 100%, *p* = 0.26). The proportion of patients who received a dose reduction in advanced, low‐ versus high‐exercise adherence also did not significantly differ for BC (22.2% vs. 16.7%, *p* = 0.72), GI (60.0% vs. 60.0%, *p* = 1.00), or PC (90.0% vs. 100%, *p* = 0.25).

### 3.6 Predictors of chemotherapy completion

Cancer site was significantly associated with RDI. Compared with BC, patients with GI (*β* = −0.12, *p* = 0.03) and PC (*β* = −0.22, *p* = 0.006) achieve lower RDI. However, no significant associations between RDI and exercise adherence (*p* = 0.11), metastatic stage (*p* = 0.25), or comorbidities (*p* = 0.41) were seen.

Several significant associations were observed (Table 4). For GI patients, every 2.72 [=exp (1)] unit increase in overall exercise adherence led to a significant 7% decrease in RDI (*p* = 0.001). There was a significant difference in RDI between metastatic and nonmetastatic patients where metastatic patients saw a 15% increase in RDI for every 2.72 unit increase in overall exercise adherence (*p* = 0.04). Additionally, for every 2.72 unit increase, the number of pre‐intervention comorbidities resulted in a 7% decrease in RDI (*p* = 0.05).

**TABLE 4:** Predictors of RDI.

	Log_overall_adherence		Metastatic versus non‐metastatic		# of comorbidities	
	Exp(estimate) ± SE	*p*‐value	Exp(estimate) ± SE	*p*‐value	Exp(estimate) ± SE	*p*‐value
Breast	1.012 ± 0.028	0.67	0.944 ± 0.108	0.62	1.013 ± 0.060	0.83
GI	0.930 ± 0.020	0.001	1.156 ± 0.079	0.04	0.929 ± 0.034	0.05
Pancreatic	1.005 ± 0.032	0.88	1.013 ± 0.114	0.91	0.978 ± 0.032	0.51

Abbreviations: GI; gastrointestinal, RDI; relative dose intensity.

## 4 DISCUSSION

This pragmatic exercise trial provides important perspective on the role of exercise during chemotherapy for patients with BC, GI, and PC. To our knowledge, our study is the first to report on the chemotherapy completion rates and the influence of exercise adherence in multiple cancer groups. We reported that BC patients had significantly higher RDIs, regardless of the need for dose reductions and delays, compared to GI and PC patients. Furthermore, we found that exercise had no effect on RDI and that the level of exercise adherence did not have a positive effect on RDI. Rather, we found that with incremental increases in exercise adherence, there are decreases in RDI in GI patients.

Our findings in BC align with the existing literature showing that BC patients routinely achieve high RDI. An early randomized controlled trial (RCT) in 242 women with BC during adjuvant chemotherapy reported nonsignificant differences in the proportion of patients who had an RDI >85% (resistance training group: 78% versus control group: 65.9%, *p* = 0.08).^17^ In a subsequent RCT of 301 women with BC during adjuvant chemotherapy, three different exercise modalities elicited RDI rates of ≥85% for 82%–88% of participants with no significant differences between group (*p* = 0.57).^18^ More recently, Mijwel et al.^19^ showed in 240 women with Stages 1–3a BC that RDI across no exercise, high‐intensity interval training plus resistance training, and high‐intensity interval training plus endurance training were comparable at 86.7%, 86.5%, and 77.8%, respectively. Our results taken with previous evidence suggests that a ceiling effect exists for subsets of BC patients (i.e., younger age, Caucasian, and higher socioeconomic status)^20^ where decreases in RDI may be attributed to toxicities that may not have sufficient evidence of being targetable by exercise such as GI distress, appetite changes, and chemotherapy‐induced peripheral neuropathy.^21^

Patients in ENACT achieved a lower RDI of 76.8% compared with previous exercise trial in GI patients. Van Vulpen et al.^22^ showed that in patients with colon cancer, supervised exercise elicited an increase in RDI of 82% versus 76% in the nonexercising control group. In the PACES trial,^23^ patients with colon cancer randomized to (1) home‐based, low‐intensity physical activity, (2) moderate‐ to high‐intensity, combined resistance and aerobic exercise, or (3) usual care showed nonsignificant differences in RDI of 87%, 92%, and 78%, respectively. The discrepancy in RDI in ENACT could be due to the inclusion of a larger variety of GI diagnoses, including colorectal and rectum, who may require more adjustments to their prescribed chemotherapy plan. However, a larger proportion of participants in the ENACT trial did not achieve a RDI ≥85% (56.2%). This is similar to the findings of van Vulpen et al. with 65% of the exercise group unable to achieve a RDI ≥85%.^22^ Our results also show that for GI patients, higher amounts of exercise may have a negative effect on RDI. It could be that the combination of aggressive chemotherapy and its related side‐effects may be too overwhelming physiologically to be countered solely by exercise and may require additional support. To our knowledge, this study is the first to report that higher exercise adherence may be a contraindication to chemotherapy completion for certain patients and that “more exercise is better” may not be the general, overarching advice to prescribe to all patients during treatment. Rather, our results may indicate that the type of exercise prescribed in ENACT might not have been the most appropriate. Although higher exercise adherence levels in certain cancer sites resulted in decreases in RDI, it is important to acknowledge that the decreases seen in RDI were not clinically significant (≥15%).^6^, ^7^ This reinforces that exercise is feasible and safe during active chemotherapy treatment and that special attention must be paid to the type and dosing of exercise.

Patients in ENACT with PC achieved significantly lower RDI compared with BC and GI, aligning with the existing evidence.^24^, ^25^ The low RDI seen by PC patients may also be due to high toxicities and greater modifications due to different individual drugs in a treatment protocol. For example, Kobayashi et al. reported RDIs for four separate agents in the FOLFIRINOX treatment protocol, which ranged from 23.4% to 76.9%, demonstrating poor adherence to the chemotherapy plan.^26^ In a recent meta‐analyses,^27^ 12 studies with a total of 300 participants were reviewed on the effects of exercise on various functional and patient‐reported outcomes with no studies reporting the inclusion of chemotherapy completion. Our results are suggestive that exercise might be valuable for improving chemotherapy tolerance in PC, but our sample size was too small to draw conclusions.

Future research should consider a multimodal intervention that address chemotherapy‐related effects, such as nausea and vomiting, reflux and eating restriction, and chemotherapy‐induced peripheral neuropathy,^28^, ^29^ that exercise alone cannot. It is possible that these types of toxicities would reduce chemotherapy dose and exercise adherence. In that situation, both the chemotherapy dose reduction and exercise adherence reduction would have the same cause, but exercise may not have any impact on these particular toxicities. Multimodal interventions may be especially important in cancers that receive more toxic and aggressive treatment regimens such as GI and PC where different interventions might need to take precedent at different stages of treatment to aid chemotherapy tolerance.

## 5 LIMITATIONS

This study has a few limitations. Our analysis is a secondary analysis of chemotherapy completion rates versus a real‐time collection of chemotherapy treatment regimens, similar to previous studies that did not include RDI as a primary outcome. Collection in real‐time would allow for more accurate data and for collection of the adverse events that alter a patient's treatment regimen. Furthermore, our findings are an aggregation of patients from three cancer sites with different chemotherapy treatment regimens. Although this was done to reflect the practicalities of working in a clinical oncology setting, future similar research should target specific treatment regimens within the different cancer sites to reduce heterogeneity.

## 6 CONCLUSION

Exercise is a multifaceted and effective supportive therapy that can lend to the success of a patient's physical and psychosocial well‐being and potentially their chemotherapy tolerance and completion. RDI is influenced by a number of factors including exercise adherence. Although increased exercise adherence may not be directly associated with increased RDI, more exercise does not pose a safety concern for patients receiving active chemotherapy treatment. Future exercise trials during active chemotherapy treatment should consider: (1) the cancer group and their concomitant treatment; (2) the specifics of exercise dosing and how to address chemotoxicities that prevent exercise; and (3) multimodal interventions to address toxicities that will work alongside exercise to aid in addressing RDI.

## AUTHOR CONTRIBUTIONS

**Melanie Potiaumpai** was involved in data curation, formal analysis, investigation, methodology, project administration, writing—original draft, and writing—review and editing. **Erica A. Schleicher** was involved in data curation, project administration, and writing—review and editing. **Ming Wang** was involved in methodology, and writing—review and editing. **Kristin L. Campbell** was involved in methodology, and writing—review and editing. **Kathleen Sturgeon** was involved in methodology, and writing—review and editing. **Kathryn H. Schmitz** was involved in conceptualization, data curation, formal analysis, investigation, methodology, project administration, supervision, writing—original draft, and writing—review and editing.

## FUNDING INFORMATION

No funding was received for this trial.

## CONFLICT OF INTEREST STATEMENT

The authors have no conflicts of interest to disclose.

## ETHICS STATEMENT

Approval was obtained from the Penn State University Institutional Review Board.

## CLINICAL TRIAL REGISTRATION NUMBER

The trial was registered at ClinicalTrials.gov (study number NCT03461471).

## Data Availability

The data are not publicly available due to privacy or ethical restrictions.

## References

[1] Siegel RL , Miller KD , Fuchs HE , Jemal A . Cancer statistics, 2021. CA Cancer J Clin. 2021;71:7‐33. 33433946 10.3322/caac.21654

[2] Halpern MT , Yabroff KR . Prevalence of outpatient cancer treatment in the United States: estimates from the medical panel expenditures survey (MEPS). Cancer Invest. 2008;26:647‐651. 18584358 10.1080/07357900801905519

[3] Wood WC , Budman DR , Korzun AH , et al. Dose and dose intensity of adjuvant chemotherapy for stage II, node‐positive breast carcinoma. N Engl J Med. 1994;330:1253‐1259. 8080512 10.1056/NEJM199405053301801

[4] Zhang L , Yu Q , Wu XC , et al. Impact of chemotherapy relative dose intensity on cause‐specific and overall survival for stage I–III breast cancer: ER+/PR+, HER2− vs. triple‐negative. Breast Cancer Res Treat. 2018;169:175‐187. 29368311 10.1007/s10549-017-4646-1 PMC6190707

[5] Qi W , Wang X , Gan L , Li Y , Li H , Cheng Q . The effect of reduced RDI of chemotherapy on the outcome of breast cancer patients. Sci Rep. 2020;10:13241. 32764734 10.1038/s41598-020-70187-8 PMC7413525

[6] Lyman GH . Impact of chemotherapy dose intensity on cancer patient outcomes. J Natl Compr Canc Netw. 2009;7:99‐108. 19176210 10.6004/jnccn.2009.0009

[7] Denduluri N , Patt DA , Wang Y , et al. Dose delays, dose reductions, and relative dose intensity in patients with cancer who received adjuvant or neoadjuvant chemotherapy in community oncology practices. J Natl Compr Canc Netw. 2015;13:1383‐1393. 26553767 10.6004/jnccn.2015.0166

[8] van Abbema DL , van den Akker M , Janssen‐Heijnen ML , et al. Patient‐ and tumor‐related predictors of chemotherapy intolerance in older patients with cancer: a systematic review. J Geriatr Oncol. 2019;10:31‐41. 29706424 10.1016/j.jgo.2018.04.001

[9] Bland KA , Zadravec K , Landry T , Weller S , Meyers L , Campbell KL . Impact of exercise on chemotherapy completion rate: a systematic review of the evidence and recommendations for future exercise oncology research. Crit Rev Oncol Hematol. 2019;136:79‐85. 30878132 10.1016/j.critrevonc.2019.02.005

[10] Kirkham AA . Supervised, multimodal exercise: the chemotherapy supportive therapy that almost does it all. Oncologist. 2020;25:3‐5. 31754070 10.1634/theoncologist.2019-0628 PMC6964116

[11] Potiaumpai M , Doerksen SE , Chinchilli VM , et al. Cost evaluation of an exercise oncology intervention: the exercise in all chemotherapy trial. Cancer Reports. 2022;5:e1490. 34236137 10.1002/cnr2.1490 PMC8955063

[12] An KY , Arthuso FZ , Kang DW , et al. Exercise and health‐related fitness predictors of chemotherapy completion in breast cancer patients: pooled analysis of two multicenter trials. Breast Cancer Res Treat. 2021;188:399‐407. 33779887 10.1007/s10549-021-06205-8

[13] Groen WG , Naaktgeboren WR , van Harten WH , et al. Physical fitness and chemotherapy tolerance in patients with early‐stage breast cancer. Med Sci Sports Exerc. 2022;54:537‐542. 34961754 10.1249/MSS.0000000000002828 PMC8920022

[14] Schmitz KH , Potiaumpai M , Schleicher EA , et al. The exercise in all chemotherapy trial. Cancer. 2021;127:1507‐1516. 33332587 10.1002/cncr.33390

[15] Longo DL , Duffey PL , DeVita VT Jr , Wesley MN , Hubbard SM , Young RC . The calculation of actual or received dose intensity: a comparison of published methods. J Clin Oncol. 1991;9:2042‐2051. 1941063 10.1200/JCO.1991.9.11.2042

[16] Weycker D , Barron R , Edelsberg J , Kartashov A , Lyman GH . Incidence of reduced chemotherapy relative dose intensity among women with early stage breast cancer in US clinical practice. Breast Cancer Res Treat. 2012;133:301‐310. 22270932 10.1007/s10549-011-1949-5

[17] Courneya KS , Segal RJ , Mackey JR , et al. Effects of aerobic and resistance exercise in breast cancer patients receiving adjuvant chemotherapy: a multicenter randomized controlled trial. J Clin Oncol. 2007;25:4396‐4404. 17785708 10.1200/JCO.2006.08.2024

[18] Courneya KS , McKenzie DC , Mackey JR , et al. Effects of exercise dose and type during breast cancer chemotherapy: multicenter randomized trial. J Natl Cancer Inst. 2013;105:1821‐1832. 24151326 10.1093/jnci/djt297

[19] Mijwel S , Bolam KA , Gerrevall J , Foukakis T , Wengstrom Y , Rundqvist H . Effects of exercise on chemotherapy completion and hospitalization rates: the OptiTrain breast cancer trial. Oncologist. 2020;25:23‐32. 31391297 10.1634/theoncologist.2019-0262 PMC6964125

[20] Bandera EV , Alfano CM , Qin B , Kang DW , Friel CP , Dieli‐Conwright CM . Harnessing nutrition and physical activity for breast cancer prevention and control to reduce racial/ethnic cancer health disparities. Am Soc Clin Oncol Educ Book. 2021;41:1‐17. 10.1200/EDBK_321315 33989021

[21] Kleckner IR , Dunne RF , Asare M , et al. Exercise for toxicity management in cancer–a narrative review. Oncol Hematol Rev. 2018;14:28‐37. 29713475 PMC5922767

[22] Van Vulpen JK , Velthuis MJ , Steins Bisschop CN , et al. Effects of an exercise program in colon cancer patients undergoing chemotherapy. Med Sci Sports Exerc. 2016;48:767‐775. 26694846 10.1249/MSS.0000000000000855

[23] van Waart H , Stuiver MM , van Harten WH , et al. Recruitment to and pilot results of the PACES randomized trial of physical exercise during adjuvant chemotherapy for colon cancer. Int J Colorectal Dis. 2018;33:29‐40. 29124329 10.1007/s00384-017-2921-6

[24] Fernandez A , Salgado M , Garcia A , et al. Prognostic factors for survival with nab‐paclitaxel plus gemcitabine in metastatic pancreatic cancer in real‐life practice: the ANICE‐PaC study. BMC Cancer. 2018;18:1185. 30497432 10.1186/s12885-018-5101-3 PMC6267080

[25] Ishigaki K , Ozaka M , Kataoka S , et al. The relationship between antitumor effects and relative dose intensity of gemcitabine plus nab‐paclitaxel for unresectable pancreatic cancer. J Clin Oncol. 2017;35:419.

[26] Kobayashi S , Ueno M , Omae K , et al. Influence of initial dose intensity on efficacy of FOLFIRINOX in patients with advanced pancreatic cancer. Oncotarget. 2019;10:1775‐1784. 30956757 10.18632/oncotarget.26633 PMC6442997

[27] O'Connor D , Brown M , Eatock M , Turkington RC , Prue G . Exercise efficacy and prescription during treatment for pancreatic ductal adenocarcinoma: a systematic review. BMC Cancer. 2021;21:43. 33422020 10.1186/s12885-020-07733-0 PMC7794639

[28] Given BA , Given CW , Sikorskii A , Hadar N . Symptom clusters and physical function for patients receiving chemotherapy. Semin Oncol Nurs. 2007;23:121‐126. 17512439 10.1016/j.soncn.2007.01.005

[29] Numico G , Longo V , Courthod G , Silvestris N . Cancer survivorship: long‐term side‐effects of anticancer treatments of gastrointestinal cancer. Curr Opin Oncol. 2015;27:351‐357. 26049277 10.1097/CCO.0000000000000203

